# Guanidinoacetic acid supplementation and stocking density effects on broiler performance: behavior, biochemistry, immunity, and small intestinal histomorphology

**DOI:** 10.1186/s13028-024-00782-6

**Published:** 2024-12-18

**Authors:** Mohammad Alaa, Abeer Hamada Abdel Razek, Mohamed Ahmed Tony, Aya Mohye Yassin, Mohamad Warda, Mohamed Ahmed Awad, Basma Mohamed Bawish

**Affiliations:** 1 Department of Veterinary Hygiene and Management, Faculty of Veterinary Medicine, Cairo University, Giza, 12211 Egypt; 2 Department of Nutrition and Clinical Nutrition, Faculty of Veterinary Medicine, Cairo University, Giza, 12211 Egypt; 3 Department of Biochemistry and Molecular Biology, Faculty of Veterinary Medicine, Cairo University, Giza, 12211 Egypt; 4 Department of Animal Physiology, Faculty of Veterinary Medicine, Ataturk University, 25240 Erzurum, Turkey; 5 Department of Cytology and Histology, Faculty of Veterinary Medicine, Cairo University, Giza, 12211 Egypt

**Keywords:** Broiler chickens, GAA, Hemagglutination-inhibition test, Ingestive behavior, Oxidative stress, Performance, Stress indicators

## Abstract

**Background:**

Rearing poultry under stressful high stocking density (HSD) conditions is a common commercial practice to increase profitability, despite its negative effects on broiler physiology and welfare. Many feed additives are used to alleviate the negative impact of such practices. This study investigated the ameliorative effects of guanidinoacetic acid (GAA) on growth performance, ingestive behavior, immune response, antioxidant status, stress indicators, and intestinal histomorphometry of broilers subjected to HSD. A total of 364 male broilers were randomly allocated into four treatments with 7 replicates each in a 2 × 2 factorial arrangement: two stocking densities (SD) (10 and 16 birds/m^2^) and two GAA levels (0 and 0.6 g/kg feed).

**Results:**

Body weight, weight gain, feed intake, feed conversion ratio, production efficiency factor, dressing yield, and ingestive behavior were negatively affected by HSD, whereas the mortality rate was unaffected (*P* > 0*.*05). GAA improved the overall growth performance and dressing percentage (*P* < 0.05). In the HSD group, the immune response decreased at d 21 (*P* < 0*.*05). Creatine kinase, glutathione peroxidase (GPX), superoxide dismutase, catalase, triglycerides, and villus length and width (ileum) were reduced, whereas corticosterone (CORT) was increased (*P* < 0*.*05). Moreover, GAA increased the hemagglutination-inhibition titer at 21 days and the levels of lactate dehydrogenase, GPX, and catalase and decreased the levels of creatinine, alanine aminotransferase, nitrite, triglycerides, and CORT (*P* < 0*.*05). SD and GAA did not affect malondialdehyde or other biochemical parameters (*P* > 0*.*05).

**Conclusions:**

Dietary GAA supplementation can improve productivity and antioxidant status and reduce stress in broilers reared in a HSD environment.

## Background

A stress-free environment is the main target in poultry production. Poultry in commercial farms face various stressors, such as high stocking density (HSD), high ambient temperature, improper management, low sanitation, and disease challenges, which harm their welfare [1]. Stocking density (SD) is considered one of the major issues for the livestock industry in many countries [2]. Many welfare problems, such as behavioral changes, different kinds of diseases and disorders, and high mortality rates, have manifested in intensified rearing systems [3]. HSD negatively impacts growth performance [4, 5] and affects nutrient digestibility by reducing villi development and absorptive surface area [6]. Additionally, HSD results in significant changes in physiological stress indicators such as elevated blood stress hormones [7], high glucose and cholesterol levels [8], a decreased immune response [9], and increased oxidative stress [10]. The net negative effects of HSD can be reflected in the carcass weight and relative organ weights [11, 12]. Although the profit per chicken decreases at HSD, the total meat production per square meter of floor area increases, resulting in a greater total profit [9]. Therefore, most poultry producers follow major policies focused on rapid growth, minimum space allowances, and the lowest production cost [13].

Currently, poultry producers are focusing on reducing the negative effects of HSD to maximize profit, using feed additives such as prebiotics, symbiotics, and alpha-lipoic acid [14, 15]. Guanidinoacetic acid (GAA) is synthesized in the avian kidney from arginine and glycine amino acids and is then methylated in the liver yielding creatine [16, 17]. Creatine is an important nutrient in energy metabolism, especially in muscle cells [16, 18]. In addition to its direct function in muscle accretion, dietary GAA can “spare” arginine, in poultry corn-soybean diets, enabling more arginine for muscle regeneration and growth; in addition to its antioxidant properties [19–22].

GAA is gaining popularity in the feed industry because of its economic affordability and chemical stability during feedstuff processing [23]. Currently, GAA is an approved source of creatine in Europe and the United States [24]. GAA added to a vegetable protein-based diet promotes the performance and carcass characteristics of the broiler chickens [24]. Birds subjected to cold stress and fed a GAA-rich diet presented a reduction in lipid peroxidation as indicated by higher liver GPx and serum CK levels, lower MDA levels, and improved FCR [25]. In heat stress, the GAA supplementation; especially at a dose of 0.6 g/kg, reduces oxidative damage and improves the intestinal histomorphometry, thus preventing the negative effects of heat stress on growth and mortality [26].

In our previous work, we demonstrated the effects of GAA on several behavioral patterns (comfort and locomotor behavior) and leg health in broilers subjected to HSD [27]. However, no comprehensive study has investigated the impact of dietary GAA supplementation on the productivity, gut health, oxidative stress parameters, and immune status of broiler chickens raised under HSD. Therefore, this study investigated the effects of GAA supplementation (0.6 g/kg feed) on broiler performance, ingestive behavior, antioxidant status, stress indicators, immune response, and intestinal histomorphometry under low (10 birds/m^2^) and HSD (16 birds/m^2^) conditions.

## Methods

### Ethical approval

This study was approved by the Institutional Animal Care and Use Committee guidelines, Faculty of Veterinary Medicine, Cairo University, Egypt. (Ethical reference No: Vet CU28/4/2021/311).

### Study design

A total of 364 Arbor acres 1-day-old male broiler chicks of similar body weight (42 g) were randomly allocated via a completely randomized design with a 2 × 2 factorial arrangement into four groups with 7 replicates each (10 birds/replicate; 70 birds/group) in each LSD group, and (16 birds/replicate; 112 birds/group) in each HSD group. Group I: Birds were stocked at 10 birds/m^2^ (LSD) with a basal diet only; Group II: LSD with a basal diet supplemented with GAA (0.6 g/kg feed), Group III: high stocking density (HSD) (16 birds/m^2^) with a basal diet only; and Group IV: HSD with a basal diet supplemented with GAA (0.6 g/kg feed). The dose of GAA (CreAmino®, 96% guanidinoacetic acid (GAA), AlzChem Trostberg GmbH, Germany) [24, 28] was selected on the basis of previously published literature [27].

### Birds and housing

The experimental birds were reared in the poultry research unit of the Veterinary Hygiene and Management Department, Faculty of Veterinary Medicine, Cairo University, Egypt, for 35 days. The birds were housed in clean disinfected identical pens (1 m^2^/replicate) with 7 cm deep wood shaving litter. The temperature ranged from 32–33 °C during the first week, then decreased by 2.8°C/week until reaching 24°C, after which it was maintained until the end of the study. The RH ranged from 45 to 65%. The lighting program lasted 24 L hours from 1 to 3 days and 23 L: 1 D hours until the end of the experiment. The experimental chicks were vaccinated against Newcastle disease virus (NDV) and infectious bronchitis (IB) on day 7 of age and against infectious bursal disease virus (IBDV) on day 14 of age, and the vaccination against Newcastle disease was repeated on day 21 of age.

### Experimental diets

There are two dietary treatments (basal and experimental diets). The basal diet of three stages was used for each of the un-supplemented groups and formulated to be iso-nitrogenous to meet or marginally exceed the nutrient requirements of the other nutrients stated in the manual of the Arbor Acres broiler breed [29]. The basal diets based on corn-soybean meal presented average metabolizable energy (AME) values of 2900, 3000, and 3100 kcal/kg for the starter, grower, and finisher stages, respectively. The experimental diets were supplemented with GAA at 0.6 g/kg feed, and the AME was reduced by 80 kcal/kg for each stage. Starter diets in the form of crumbles were offered during the first 10 days of age. The grower and finisher diets were in pellets and provided for 18 and 7 days, respectively. Birds in all groups had free access to feed and water throughout the experiment.

### Growth performance measurements

Chicks were individually weighed at the time of arrival and weekly using a digital balance to obtain body weight (BW). The body weight gain (BWG), feed intake (FI), and feed conversion ratio (FCR) were calculated weekly and for the entire period of the trial (from 1 to 35 days of age) after mortality was adjusted as previously described [30]. The number of dead birds in each treatment was recorded daily to calculate the mortality rate throughout the experimental period. European Production Efficiency Factors (EPEFs) were calculated according to the following formula: EPEF = (liveability × live weight (kg)/(age in days × FCR) × 100 [31].

### Carcass traits

At the end of the experiment on day 35, the birds were starved overnight, and seven birds from each treatment were randomly selected and weighed (live BW) via a digital balance. The processes of slaughtering, bleeding, scalding, defeathering, and evisceration (dressed carcass) were performed according to standard procedures. The carcass was weighed, and the dressing percentage was calculated according to the following equation [32]:

$${\text{Dressing percentage}}\,\left( \% \right) = \left( {\text{Dressed weight}} \right)/\left( {\text{Preslaughter live weight}} \right) \times 100$$

Breast muscle and leg weights, including thigh and drumstick weights, were weighed and expressed as a percentage of BW. Additionally, the liver, heart, gizzard, proventriculus, abdominal fat, and immune organs (spleen, bursa of Fabricius, and thymus) were weighed, and the relative weights were measured [33, 34].

### Ingestive behavior

The ingestive behavior of broiler chickens (feeding and drinking) was recorded using instantaneous scan sampling from 2 to 5 weeks of age (end of the experimental period) as mentioned previously [27]. A bird pecking in the feeder is considered feeding behavior, whereas a bird pecking in the drinker is considered drinking behavior [35]. The behavior occurrence proportion of each behavior is calculated from the sum of the observed behaviors.

### Biochemical analysis

#### Sample preparation

Seven chicks from each group were randomly selected (d 35), and blood samples were collected after slaughter into plain, K2 EDTA, and sodium fluoride tubes. The serum and plasma were separated via blood centrifugation at 3500 rpm for 15 min and stored at − 80 °C until analysis. Liver samples were taken for evaluation of the antioxidant status. The samples were homogenized in 100 mM potassium phosphate, pH 7, containing 2 mM EDTA/g tissue for assessment of SOD activity and nitrite concentrations, whereas for GPx activity, the liver was homogenized in 50 mM phosphate buffer, pH 7, containing 5 mM EDTA and 1 mM 2-mercaptoethanol. The homogenates were centrifuged at 4000 rpm for 15 min at 4 °C; the supernatants were collected and stored at − 80 °C until analysis [36].

#### Blood biochemical indices and lipid profile

The serum samples were analyzed spectrophotometrically (UV-2100 spectrophotometer, USA) for total protein (546 nm), albumin (578 nm), uric acid (546 nm), creatinine (492 nm), alanine aminotransferase (ALT) (546 nm), aspartate aminotransferase (AST) (340 nm), and plasma glucose (546 nm) levels via commercial kits (BioDiagnostic, Giza, Egypt). Blood lipid profiles (serum cholesterol, triglyceride (TAG), high-density lipoprotein (HDL), and low-density lipoprotein (LDL) (546 nm) were determined via reagent kits according to the manufacturer’s guidelines (Spectrum Company, Cairo, Egypt).

#### Energy-related variable measurements

The serum concentrations of energy-related enzymes (lactate dehydrogenase) (LDL) and creatine kinase (CK) activities (340 nm) were kinetically assayed using commercial diagnostic kits (Centronics GmbH, Wartenberg, Germany) according to the manufacturer's instructions.

#### Determination of the antioxidant status

Plasma catalase (CAT) activity was assayed spectrophotometrically (510 nm) via a commercial kit (Bio Diagnostic, Giza, Egypt) [37]. Liver SOD and GPX (340 nm) activities (U/gT) were determined kinetically via commercial kits (Bio Diagnostic, Giza, Egypt) according to the methods of [38, 39], respectively.

#### Determination of oxidative stress biomarkers

The tissue nitrite concentration was determined through measurement of the concentration of its final product, nitrite, according to [40]. One hundred microliters of the tissue homogenate was added to 100 µL of Griess reagent, which converts nitrite into a deep purple azo compound, and the absorbance was measured at 540 nm via a spectrophotometer. The serum malondialdehyde (MDA) concentration was used as an index of lipid peroxidation as described previously [41]. The MDA content was determined by measuring the levels of thiobarbituric acid reactive species. The absorbance of the resulting pink product was measured spectrophotometrically at 534 nm.

#### Evaluation of corticosterone (CORT) levels

Serum CORT levels were measured via a chicken CORT ELISA kit (catalog no: SG-80021; Sino Gene Clon Biotech Co., Ltd., Hangzhou, China) according to the manufacturer’s instructions. The optical density of the samples was recorded at a wavelength of 450 nm via a microplate reader (ELx800™ Absorbance Readers, BioTek Instruments, Inc., Vermont, USA). The sample concentration was calculated through the straight-line regression equation of the standard curve of the standard concentration and the OD value, with the sample OD value in the equation.

#### Antibody titer against Newcastle disease

Antibody titer determination via the hemagglutination inhibition test (HI) was carried out to evaluate the immune response of seven broiler chickens at d 21 from the wing vein and at d 35 (at slaughter) against the NDV vaccine in serum samples [42]. Twofold serial dilutions of the serum samples were carried out. Four hemagglutination units (HAUs) of attenuated NDV (Lasota) commercial antigens were prepared. A 1% suspension of chicken erythrocytes was used. The results are expressed as the mean log2 HI titers.

#### Histomorphometric evaluation of intestinal villi

At the end of the experimental period, seven birds from each group were slaughtered, defeathered, and eviscerated to collect the samples for histomorphometry evaluation. The specimens (small intestine; duodenum, jejunum, and ileum) were collected, fixed in 10% neutral buffered formalin (10% NBF), washed, dehydrated, cleared, and embedded in paraffin blocks. Then, the sections (4 μm thick) were sectioned via a microtome (Leica, Germany) for hematoxylin and eosin (H&E) staining [43]. The stained slides were viewed via a light microscope (Leica DM500) at × 200 and  × 400 magnification, and then images were captured with a Leica ICC50 HD camera attached to the microscope and finally examined and analyzed via image analysis software (Leica Microsystems (LAS version 3.8.0 [build:878] Leica Ltd.) image analyzer computer system). The following morphometric measurements were taken: the villus height (μm), which was measured from the tip to the base of the villus, and the villus width (μm), which was measured at three points: the apex, middle, and base. The ratio of villus height: villus width [44] and the absorption surface area (ASA) was also calculated according to the [45] formula, adapted as follows: ASA = (width of the folds × height of the folds)/(width of the folds/2)^2^ (μm^2^) [46].

### Statistical analysis

The data were checked for normality via Shapiro‒Wilk tests and for homogeneity of variance via Levene’s test. All the data were analyzed via 2-way ANOVA in a completely randomized design via PASW Statistics, version 24.0 software (SPSS Inc., Armonk, NY, USA). The statistical model included the main effects of SD and GAA level and their interaction. The results are reported as the means and standard errors of the means (SEMs). Tukey’s post hoc test was used for multiple comparisons. The data were considered significantly different at *P* < 0*.*05. GraphPad Prism version 6.00 was used to create graphs to compare the means ± SEs of the different groups (GraphPad Software, San Diego, CA, USA). *P* < 0.05 was considered to indicate statistical significance.

## Results

### Productive performance

The effects of different stocking densities (10 and 16 birds/m^2^) and dietary supplementation with GAA (0.6 g/kg feed) on the growth performance parameters of broiler chickens and their interactions are shown in Tables 1, 2 and 3. A high stocking density (HSD) harms the growth performance. Increasing the stocking density from 10 to 16 birds/m^2^ significantly (*P* < 0*.*05) reduced the body weight (BW) and feed intake (FI) at days 14, 21, 28, 35, and during the cumulative period in the HSD group compared with those in the LSD group. Additionally, HSD significantly (*P* < 0*.*05) decreased body weight gain (BWG) on day 14 and during the cumulative period, with a numerical decrease at days 21, 28, and 35 compared with that in the LSD group. Moreover, the European Production Efficiency Factor (EPEF) was lower in the HSD group than in the LSD group (*P* < 0*.*05). The worst feed conversion ratio (FCR) was observed in the HSD group. Additionally, it was numerically greater at days 28, 35, and during the cumulative period than in the LSD group. However, there was no significant difference in mortality between the two groups (*P* > 0*.*05).

**Table 1:** Efficacy of GAA supplementation on BW and BWG of broilers reared under HSD conditions

Stocking density (birds/m^2^)	GAA (g/kg feed)	Average body weight (g)					Average body weight gain (g)				
		D 7	D 14	D 21	D 28	D 35	D 7	D 14	D 21	D 28	D 35
LSD	0	207.00	563.29^a^	1149.75^b^	1911.29^b^	2649.86^b^	165.00	356.27^a^	586.29^ab^	761.72^b^	738,57^ab^
LSD	0.6	204.43	564.00^a^	1172.71^a^	1969.71^a^	2741.14^a^	159.29	359.57^a^	608.71^a^	797.00^a^	771.43^a^
HSD	0	204.57	526.86^b^	1075.57^d^	1811.29^d^	2507.57^d^	159.43	322.29^b^	548.71^b^	735.71^b^	696.28^b^
HSD	0.6	206.43	542.00^ab^	1119.86^c^	1866.86^c^	2588.29^c^	161.29	335.57^ab^	577.86^ab^	747.00^b^	721.43^b^
Pooled SEM		2.24	4.98	7.52	11.51	17.22	1.19	4.94	6.66	6.12	7.42
*P* value											
SD		0.964	**0.002**	** < 0.001**	** < 0.001**	** < 0.001**	0.455	**0.002**	**0.005**	**0.001**	** < 0.001**
GAA		0.940	0.357	** < 0.001**	** < 0.001**	** < 0.001**	0.421	0.336	**0.029**	**0.015**	**0.015**
SD × GAA		0.643	0.401	0.087	0.803	0.630	0.121	0.559	0.764	0.192	0.731

*P* value in bold are statistically significant

LSD, low stocking density (10 birds/m^2^); HSD, high stocking density (16 birds/m^2^); SD, stocking density; GAA, guanidinoacetic acid (0.6 g/kg) feed

^a,b,c,d^Different superscript letters in the same column indicate a significant difference (*P < *0.05). Pooled SEM: total SEM

**Table 2:** Efficacy of GAA supplementation on WFI and FCR of broilers reared under HSD conditions

Stocking density (birds/m^2^)	GAA (g/kg feed)	Weekly Feed intake (g)					Feed conversion ratio (g/g)				
		D 7	D 14	D 21	D 28	D 35	D 7	D 14	D 21	D 28	D 35
LSD	0	163.57	469.29^a^	723.29^a^	962.86^a^	1194.86^a^	1.04	1.32	1.24	1.26^ab^	1.62^ab^
LSD	0.6	160.29	448.57^ab^	718.00^ab^	959.57^ab^	1189.43^ab^	1.01	1.25	1.18	1.21^b^	1.54^b^
HSD	0	167.00	437.43^b^	693.14^b^	940.71^bc^	1163.86^b^	1.05	1.36	1.26	1.28^a^	1.66^a^
HSD	0.6	164.29	432.43^b^	709.14^ab^	937.14^c^	1166.29^b^	1.02	1.29	1.23	1.26^ab^	1.62^ab^
Pooled SEM		1.31	4.06	3.79	4.00	4.88	0.01	0.02	0.01	0.01	0.02
*P* value											
SD		0.166	**> 0.001**	**0.007**	**0.004**	**0.006**	0.639	0.186	0.096	**0.048**	**0.032**
GAA		0.260	0.055	0.422	0.634	0.821	0.184	**0.026**	**0.048**	**0.013**	**0.044**
SD × GAA		0.913	0.230	0.118	0.984	0.617	0.949	0.988	0.642	0.287	0.526

*P* value in bold are statistically significant

WFI, weekly feed intake; FCR, feed conversion ratio; LSD, low stocking density (10 birds/m^2^); HSD, high stocking density (16 birds/m^2^); SD, stocking density; GAA, guanidinoacetic acid (0.6 g/kg) feed

^a,b,c^Different superscript letters in the same column indicate a significant difference (*P < *0.05). Pooled SEM: total SEM

**Table 3:** Efficacy of GAA supplementation on the cumulative growth performance of broilers reared under HSD (d 1–35)

Stocking density (birds/m^2^)	GAA (g/kg feed)	BW (g)	BWG (g)	FI (g)	FCR (g/g)	Mortality (%)	EPEF
LSD	0	2649.86^b^	2607.86^b^	3518.57^a^	1.34^ab^	7.14	521.02^b^
LSD	0.6	2741.14^a^	2699.14^a^	3474.86^ab^	1.29^c^	4.29	582.16^a^
HSD	0	2507.57^d^	2469.14^d^	3402.14^b^	1.38^a^	8.92	474.38^c^
HSD	0.6	2588.29^c^	2546.61^c^	3409.29^b^	1.34^b^	7.14	513.36^bc^
Pooled SEM		17.22	17.01	13.16	0.01	0.81	8.81
*P* value							
SD		** < 0.001**	** < 0.001**	** < 0.001**	** < 0.001**	0.153	**< 0.001**
GAA		** < 0.001**	** < 0.001**	0.366	** < 0.001**	0.153	**< 0.001**
SD × GAA		0.630	0.522	0.212	0.199	0.736	0.280

*P *value in bold are statistically significant

LSD, Low stocking density; HSD, High stocking density; SD, Stocking density; GAA, Guanidinoacetic acid; BW, body weight; BWG, body weight gain; FI, feed intake; FCR, feed conversion ratio (g of feed/g of weight gain); EPEF, European Production Efficiency Factor = (livability × live weight (kg)/(age in days × FCR) × 100

^a,b,c,d^Different superscript letters in the same column indicate a significant difference (*P < *0.05), Pooled SEM: total SEM

Dietary GAA (0.6 g/kg feed) markedly improved the growth performance parameters in our experimental research. Concerning the low stocking density (LSD) conditions, the BW in the LSD + GAA group was significantly (*P* < 0*.*05) greater at days 21, 28, 35, and during the cumulative period compared with that in the LSD group. The BWG was markedly improved in LSD + GAA on day 28 and during the cumulative period (*P* < 0*.*05), with a numerical increase at days 21 and 35 compared with that in the LSD group. The FCR enhanced at days 28 and 35 with a significant improvement in the overall period (*P* < 0*.*05) in the LSD + GAA group compared with that in the LSD group. Additionally, greater EPEF was observed in the LSD + GAA group than in the LSD group. Although there was a reduction in the cumulative FI and mortality rate between the LSD + GAA and LSD groups, no significant difference was observed between them.

Regarding the HSD conditions, the dietary GAA in the HSD + GAA group showed greater live BW at days 14, 21, 28, 35, and during the cumulative period compared with that in the HSD group (*P* < 0*.*05). Moreover, The BWG improved at days 14, 21, and during the cumulative period (*P* < 0*.*05) compared with that in the HSD group. The FCR was markedly enhanced at days 28, 35, and during the cumulative period with an improvement in EPEF compared with that in the HSD group. However, no difference in mortality rate and the FI was observed between the two groups.

### Carcass traits

As shown in Table 4, the highest dressing yield was observed in the LSD + GAA group, while the lowest yield was observed in the HSD group compared with the other groups (*P* < 0*.*05), Moreover, the HSD + GAA group showed a substantial increase in the dressing yield compared with the HSD group. SD and GAA supplementation did not significantly (*P* > 0*.*05) affect the immune organs and giblet weights (except the liver) of the broiler chickens. There was a significant decrease (*P* < 0*.*05) in relative liver weight in the LSD + GAA group compared with the LSD group, whereas no difference between the HSD + GAA and HSD groups.

**Table 4:** Efficacy of GAA supplementation on carcass traits of broilers reared under HSD conditions

Stocking density (birds/m^2^)	GAA (g/kg feed)	Dressing (%)	Breast (%)	Leg (%)	Crop (%)	Provent. (%)	Gizzard (%)	Liver (%)	Heart (%)	Abd. fat (%)	Spleen (%)	Thymus (%)	Bursa (%)
LSD	0	74.72^ab^	26.42	19.52	0.36	0.30	0.97	2.54^a^	0.54	1.18	0.13	0.52	0.20
LSD	0.6	78.08^a^	27.29	20.20	0.39	0.28	1.07	2.01^b^	0.57	1.39	0.14	0.47	0.22
HSD	0	73.96^b^	25.79	20.06	0.35	0.27	0.99	2.29^ab^	0.48	1.29	0.13	0.46	0.18
HSD	0.6	75.83^ab^	26.19	19.72	0.37	0.32	1.10	2.29^ab^	0.47	1.58	0.12	0.49	0.24
Pooled SEM		0.69	0.38	0.31	0.01	0.01	0.03	0.06	0.02	0.09	0.004	0.03	0.01
*P* value													
SD		0.217	0.267	0.965	0.618	0.709	0.712	0.903	0.056	0.412	0.185	0.835	0.893
GAA		**0.045**	0.413	0.791	0.462	0.494	0.140	**0.034**	0.855	0.163	0.457	0.905	0.198
SD × GAA		0.503	0.766	0.432	0.795	0.089	0.947	**0.031**	0.545	0.838	0.373	0.746	0.420

*P* value in bold are statistically significant

LSD, low stocking density (10 birds/m^2^); HSD, high stocking density (16 birds/m^2^); SD, stocking density; GAA, guanidinoacetic acid (0.6 g/kg) feed; Prevent., Proventriculus; Abd. Fat, Abdominal fat

^a,b^Different superscript letters in the same column indicate a significant difference (*P < *0.05), Pooled SEM: total SEM

### Ingestive behavior

**Fig. 1:**
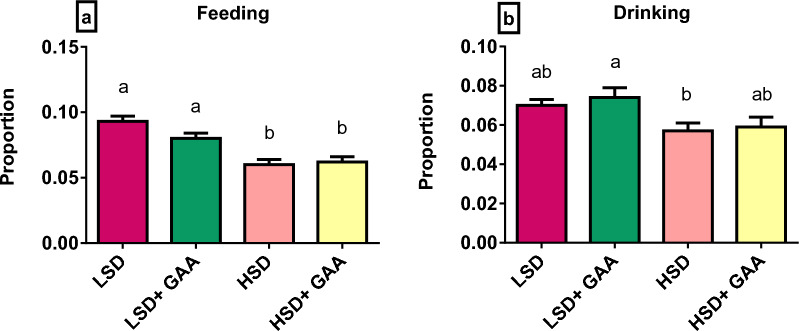
Feeding and drinking behavior of broiler chickens. Values are expressed as the mean proportion and standard error of **a** Feeding behavior and (**b)** Drinking behavior. LSD: low stocking density (10 birds/m^2^) + basal diet only, HSD: high stocking density (16 birds/m^2^) + basal diet only, LSD + GAA: low stocking density + (basal diet + GAA (0.6 g/kg) feed), HSD + GAA: high stocking density + (basal diet  + GAA (0.6 g/ kg) feed). ^a,b^Indicates statistical significance (*P* < 0.05)

Figure 1 presents the effects of different stocking densities and GAA supplementation on the ingestive behavior (feeding and drinking proportion) of broiler chickens. The HSD group showed a significant decrease (*P* < 0*.*05) in the feeding and drinking proportions compared with the LSD group. Meanwhile, in relation to the GAA supplementation, no difference in feeding and drinking behavior was detected between the LSD + GAA and LSD groups under the LSD conditions, and between HSD + GAA and HSD groups under the HSD conditions (*P* > 0*.*05).

### Antibody titer against Newcastle disease

**Fig. 2:**
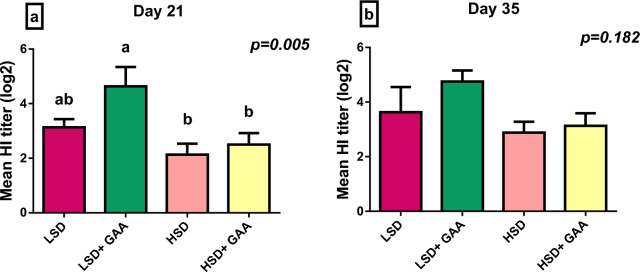
Antibody titers after NDV vaccination in the sera of broiler chickens (days 21 and 35). Values are expressed as the mean HI titer (log 2) and standard error at **a** Day 21 and **b** Day 35. LSD: low stocking density (10 birds/m^2^) + basal diet only, HSD: high stocking density (16 birds/m^2^) + basal diet only, LSD + GAA: low stocking density + (basal diet + GAA (0.6 g/kg) feed), HSD + GAA: high stocking density + (basal diet + GAA (0.6 g/kg) feed) . ^a,b^Indicates statistical significance (*P* < 0.05)

The results of mean haemagglutination inhibition (HI) titers against NDV vaccination for the tested groups are displayed in Fig. 2. On day 21, the highest mean of HI titer was observed in the LSD + GAA group, while the lowest mean was noted in the HSD group (*P* < 0*.*05). However, HI titer of the HSD + GAA group was substantially greater than that of the HSD group but the difference was not significant. On the other hand, on day 35, the mean HI antibody titer was not significantly affected by SD or GAA supplementation (*P* > 0*.*05).

### Blood biochemical parameters and lipid profile

**Fig. 3:**
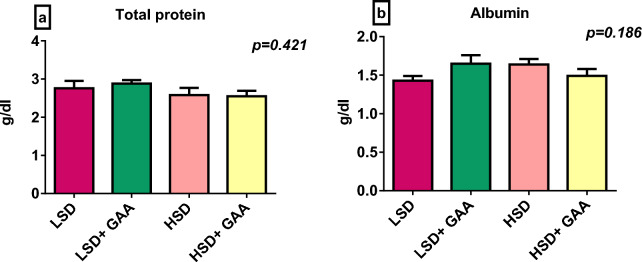
Serum total protein and albumin levels of broiler chickens. Values are expressed as the mean and standard error of **a** total protein and **b** albumin levels. LSD: low stocking density (10 birds/m^2^) + basal diet only, HSD: high stocking density (16 birds/m^2^) + basal diet only, LSD+GAA: low stocking density + (basal diet + GAA (0.6 g/kg) feed), HSD + GAA: high stocking density + (basal diet + GAA (0.6 g/kg) feed)

Blood levels of albumin, total protein, glucose, uric acid, and AST were not influenced by SD or GAA supplementation (*P* > 0*.*05) (Table 5, Fig. 3). In terms of the creatinine level and ALT activity, the HSD group did not significantly differ from the LSD group. However, GAA supplementation had a significant effect (*P* < 0*.*05) on the creatinine level between the HSD + GAA and HSD groups under the HSD condition,  while ALT was considerably varied between the LSD + GAA and LSD groups under the LSD condition (Table 5).

**Table 5:** Efficacy of GAA supplementation on some blood biochemical parameters of broilers reared under HSD conditions

Stocking density (birds/m^2^)	GAA (g/kg feed)	Glucose (mg/dl)	Uric acid (mg/dl)	Creatinine (mg/dl)	AST (U/L)	ALT (U/L)
LSD	0	213.12	5.36	0.43^ab^	291.01	7.84^a^
LSD	0.6	232.87	4.64	0.35^b^	294.54	6.33^b^
HSD	0	229.74	4.74	0.49^a^	289.26	6.91^ab^
HSD	0.6	249.53	4.14	0.39^b^	298.61	6.35^b^
Pooled SEM		5.37	0.21	0.01	5.73	0.23
*P* value						
SD		0.174	0.122	0.064	0.923	0.301
GAA		0.190	0.090	**0.002**	0.591	**0.022**
SD × GAA		0.907	0.770	0.593	0.808	0.281

*P* value in bold are statistically significant

LSD, low stocking density (10 birds/m^2^); HSD, high stocking density (16 birds/m^2^); SD, stocking density; GAA, Guanidinoacetic acid (0.6 mg/kg) feed; T. Protein, Total protein; ALT, Alanine transaminase; AST, Aspartate transaminase

^a,b^Different superscript letters in the same column indicate a significant difference (*P < *0.05). Pooled SEM: total SEM

**Fig. 4:**
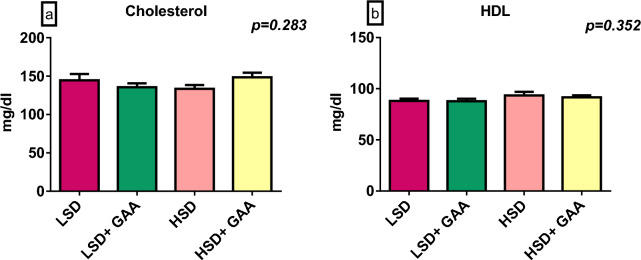
Serum cholesterol and high-density lipoprotein (HDL) levels of broiler chickens. Values are expressed as the mean and standard error of **a** cholesterol (SDs) and **b** HDL levels. LSD: low stocking density (10 birds/m^2^) + basal diet only, HSD: high stocking density (16 birds/m^2^) + basal diet only, LSD + GAA: low stocking density + (basal diet + GAA (0.6 g/kg) feed), HSD + GAA: high stocking density + (basal diet + GAA (0.6 g/ kg) feed)

**Fig. 5:**
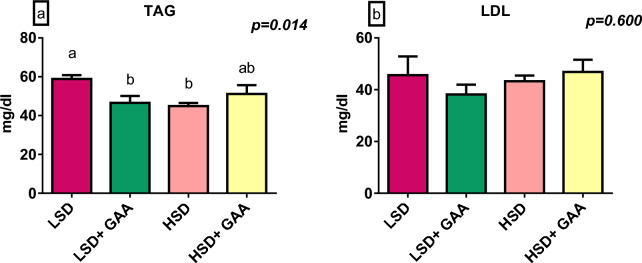
Serum triacylglycerol (TAG) and low-density lipoprotein (LDL) levels of broiler chickens. Values are expressed as the mean and standard error of **a** TAG and **b** LDL levels. LSD: low stocking density (10 birds/m^2^) + basal diet only, HSD: high stocking density (16 birds/m^2^) + basal diet only, LSD + GAA: low stocking density + (basal diet + GAA (0.6 g/kg) feed), HSD + GAA: high stocking density + (basal diet + GAA (0.6 g/kg) feed). ^a,b^Different superscript letters indicate a significant difference (*P < *0.05)

The blood lipid profiles (HDL, LDL, and cholesterol) were not significantly (*P* > 0*.*05) affected by GAA supplementation or SD. There was a significant difference (*P* < 0*.*05) in the TAG level between the HSD and LSD groups. Additionally, GAA supplementation reduced the TAG level between the LSD + GAA and LSD groups under the LSD condition (Figs. 4, 5).

### Energy-related variables

Creatine kinase (CK) activity was significantly greater in the LSD groups than in the other groups (*P* < 0*.*05), whereas GAA supplementation had no effect on CK activity (*P* > 0*.*05) (Table 6). LDH was not significantly affected by SD (*P* > 0*.*05), whereas GAA supplementation significantly increased LDH activity compared with that in the groups that received the basal diet (*P* < 0*.*05) (Table 6).

**Table 6:** Efficacy of GAA supplementation on energy-related variables, oxidant/antioxidant status parameters, and stress-related biomarkers

Stocking density (birds/m^2^)	GAA (g/kg feed)	LDH (U/L)	CK (U/L)	SOD (U/gT)	GPx (U/gT)	Catalase (U/L)	MDA (nM/ml)	Nitrite (µmol/L)	CORT (ng/ml)
LSD	0	2725.15^a^	35,515.33^a^	2024.21^a^	68.12^b^	221.20^c^	3.67	26.05^a^	3.88^c^
LSD	0.6	5622.88^b^	36,619.16^a^	2089.24^a^	120.95^a^	565.18^a^	2.92	16.30^b^	2.33^d^
HSD	0	2807.77^a^	31,183.49^b^	1793.58^b^	63.44^b^	242.28^c^	3.33	25.37^a^	7.24^a^
HSD	0.6	5636.39^b^	30,707.95^b^	1889.87^ab^	89.10^b^	372.56^b^	3.43	20.07^b^	5.23^b^
Pooled SEM		281.46	1199.31	34.07	5.55	27.21	0.14	0.87	0.32
*P* value									
SD		0.888	**0.035**	**0.001**	**0.016**	**0.015**	0.767	0.219	**< 0.001**
GAA		**< 0.001**	0.894	0.185	**< 0.001**	**< 0.001**	0.243	**< 0.001**	**< 0.001**
SD × GAA		0.920	0.738	0.795	0.069	**0.003**	0.130	0.079	0.452

*P* value in bold are statistically significant

LSD, low stocking density (10 birds/m^2^); HSD, high stocking density (16 birds/m^2^); SD, stocking density; GAA, guanidinoacetic acid (0.6 g/kg) feed; LDH, lactate dehydrogenase; CK, creatine kinase; SOD, superoxide dismutase; GPx, glutathione peroxidase; MDA, malondialdehyde; CORT, corticosterone

^a,b,c^Different superscript letters in the same column indicate a significant difference (*P < *0.05), Pooled SEM: total SEM

### Antioxidant enzyme activity

Table 6 illustrates the activity of the antioxidant enzymes. For liver GPx and plasma catalase activity, there was no difference between the HSD and LSD groups. In relation to the GAA supplementation, there was significantly higher activities in the GPx between the LSD + GAA and LSD groups, and in the catalase activity between the LSD + GAA and LSD groups in LSD conditions and between HSD + GAA and HSD groups in HSD condition (*P* < 0*.*05).

Liver SOD activity was significantly greater in the LSD group than in the HSD group (*P* < 0*.*05), whereas GAA supplementation in the LSD and HSD groups did not significantly differ*.*

### Oxidative stress biomarkers

The redox status of the broiler chickens subjected to SD and GAA supplementation treatment is presented in Table 6. Neither SD nor GAA inclusion significantly affected the serum MDA levels (*P* > 0*.*05). For the liver nitrite levels, SD had no effects on its levels*,* in contrast to the effect of GAA supplementation, which showed marked improvement proven by a significant reduction in liver nitrite level in GAA-supplemented groups compared with groups that received the basal diet only (*P* < 0*.*05).

### Corticosterone levels as stress biomarkers

As shown in Table 6, both SD and GAA supplementation significantly affected the serum CORT level. The CORT level was higher in the groups with HSD than in those with LSD (*P* < 0*.*05). The GAA-supplemented groups showed a reduction in CORT levels compared with those who received the basal diet only in LSD and HSD conditions (*P* < 0*.*05).

### Intestinal histomorphometry

**Fig. 6:**
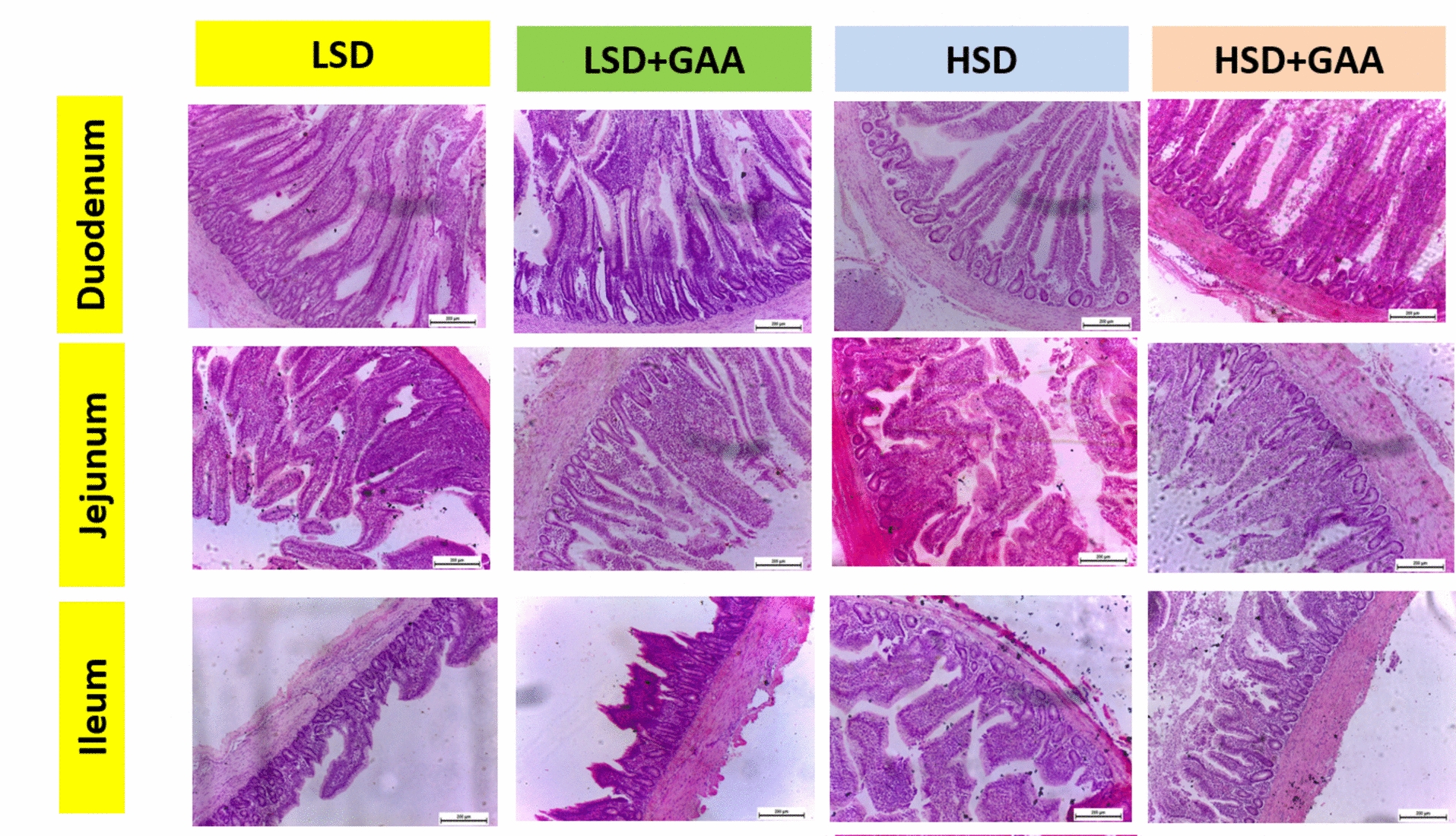
Photomicrographs of duodenum, jejunum, and ileum of broiler chickens treated with 2 × 2 factorial management. LSD: low stocking density with basal diet only . LSD + GAA: low stocking density with (0.6 g/kg feed) GAA. HSD: high stocking density with basal diet only.  HSD + GAA: high stocking density with GAA (0.6 g/kg feed). (H&E, small intestine; 4×, scale bar 200 mm)

Our findings revealed that HSD significantly reduced the villi length and width in the ileum, as observed between the HSD and LSD groups (*P* < 0*.*05), whereas the villus height and width in the duodenum and jejunum were not significantly affected by SD (*P* > 0.05). However, GAA inclusion did not markedly affect villus length or width in different intestinal parts between the different groups under LSD and HSD conditions (Table 7) (Fig. 6).

**Table 7:** Efficacy of GAA supplementation on small intestine morphological characteristics of broilers reared under HSD conditions

	GAA (g/kg feed)	Duodenum				Jejunum				Ileum			
		VL. D (μm)	VW. D (μm)	L: W ratio. D	ASA. D (mm^2^)	VL. J (μm)	VW. J (μm)	L: W ratio. J	ASA. J (mm^2^)	VL. I (μm)	VW. I (μm)	L: W ratio. I	ASA. I (mm^2^)
lSD	0	1219.70	127.76	9.87	39.47	874.24	109.68	8.01	32.06	494.65^a^	147.28^a^	3.36	16.16
LSD	0.6	1307.26	134.47	10.03	40.11	782.14	111.69	7.25	28.99	468.42^a^	135.95^ab^	3.45	14.54
HSD	0	1187.94	140.52	8.46	33.85	882.16	130.98	7.02	28.07	412.04^b^	116.27^b^	3.54	12.85
HSD	0.6	1174.23	138.22	8.87	35.47	636.72	105.09	6.25	24.98	408.89^b^	121.38^ab^	3.37	11.42
Pooled SEM		49.34	4.26	0.49	1.97	43.34	3.89	0.44	1.77	35.59	6.59	0.31	1.26
*P value*													
SD		0.429	0.359	0.217	0.217	0.414	0.317	0.279	0.279	**0.041**	**0.015**	0.763	0.763
GAA		0.722	0.805	0.782	0.782	0.052	0.110	0.402	0.402	0.152	0.413	0.363	0.363
SD × GAA		0.626	0.615	0.904	0.904	0.363	0.064	0.998	0.998	0.354	0.352	0.425	0.425

*P* value in bold are statistically significant

VL. D, villus length of duodenum; VW. D, villus width of duodenum; L: W ratio. D, length: width ratio of duodenum; ASA. D, absorptive surface area of the duodenum; VL. J, villus length of jejunum; VW. J, villus width of jejunum; L: W ratio. J, length: width ratio of jejunum; ASA. J, absorptive surface area of jejunum; VL. I, villus length of ileum; VL. I, villus width of ileum; L: W ratio. I, length: width ratio of ileum; ASA. I, absorptive surface area of the ileum; LSD, low stocking density (10 birds/m^2^); HSD, high stocking density (16 birds/m^2^); SD, stocking density; GAA, guanidinoacetic acid (0.6 g/kg) feed

^a,b^Different superscript letters in the same column indicate a significant difference (*P < *0.05), Pooled SEM: total SEM

## Discussion

Guanidinoacetic acid (GAA) is a crucial precursor of creatine in the body, assisting in energy metabolism by regulating cellular ATP homeostasis [47]. Previous studies have demonstrated the positive impact of GAA supplementation on broiler productivity and antioxidant status under both normal and stressful conditions [24–26]. However, no reports on these aspects have been published for HSD. Therefore, we hypothesized that GAA supplementation may alleviate the negative impacts of HSD on broiler performance and health status. The results of the present study revealed that HSD impaired broiler performance by decreasing BW and BWG, with simultaneous reductions in weekly FI, and similar findings were reported previously [48, 49]. Additionally, the FCR was negatively affected by the HSD, and these results were in accordance with [50, 51]. The previous results clearly revealed that LSD (10 birds/m^2^) provided the optimum environmental circumstances for chicks, allowing them to consume feed with less anxiety and thus better BW, BWG, and FCR.

The EPEF is commonly used to assess the overall economic performance of the poultry industry [52]. The lowest EPEF in our trial was found in the HSD group compared with the LSD group as previously mentioned [15]. The increased number of birds per unit area reduced the growth performance possibly due to the reduced airflow around the bird, resulting in poor FCR [2]. Overcrowding conditions lead to heat stress owing to reduced heat dissipation from birds, resulting in lower performance [53]. Heat-stressed birds use more energy in response to stressors, leaving less energy for growth [50]. Other possible explanations for poor growth in HSD-reared broilers may be reduced villus length and width, altered gut microbiota, and impaired intestinal microarchitecture [4]. No adverse effects of HSD on the mortality of broilers were noted in our results, as reported previously [54].

Our hypothesis is that GAA enhances dietary energy utilization in broilers, even under stressful conditions, as observed by improvements in BW, BWG, FCR, and EPEF [28, 55]. However, GAA had no significant effect on the weekly FI throughout the trial period [56]. The enhancement of FCR and growth in broilers supplemented with GAA was attributed to the arginine-sparing effect, enabling arginine availability for protein synthesis and muscle size augmentation [57, 58]. Additionally, owing to the increased muscle growth and ATP requirements during the late stages of broiler life, creatine or its precursor (GAA) is particularly important for replenishing the tissue creatine load [59], leading to improved energy availability and dietary nutrient utilization and growth [60]. Adding 0.6 and 1.2 g/kg GAA to broiler diets improved the final BW and overall ADG while decreasing FCR in heat-stressed broilers [26]. Moreover, Dietary GAA (0.6 g/kg) improved the feed intake, weight gain, and growth performance of broiler chickens [61]. On the other hand, the dietary inclusion of 1.2 g/kg GAA did not significantly impact the performance of broilers exposed to cold stress [25].

Our behavioral results revealed significant effects of SDs on feeding and drinking behavior, which may be related to the decrease in FI of the HSD in our performance results. Overcrowding conditions negatively impact broilers' feeding and drinking behavior due to the physical limitations in access to feeders and drinkers [53, 62]. No prior research has been done on how GAA affects broiler chicken' ingestive behavior. However, the non-significant difference in feeding and drinking behavior may be connected to the non-significant difference in the FI parameters in our study.

The dressing percentage significantly decreased with the HSD, and these results are in agreement with those of [63, 64], who reported that increasing the SD harms the dressing yield in Muscovy ducks and broilers. These results may be related to the lower final live weight of the HSD group. GAA significantly increased the dressing yield in the supplemented group because the increase in phosphocreatine in muscle cells resulted in increased dressing yield [16]. Creatine, creatine phosphate/ADP, and ATP are crucial for energy transmission in living cells, with GAA being a unique creatine precursor primarily found in muscle cells for growth and contraction [65]. An improvement in muscle mass and yield by creatine supplementation was also reported by [66]. In addition to improving the synthesis of creatine, GAA administration appears to boost skeletal muscle development via microRNA-induced upregulation of the AKT/mTOR/S6K signaling pathway [67], a crucial modulator that maintains skeletal muscle mass. Additionally, dietary arginine is spared by GAA [68], presumably allowing muscle cells to use it for protein synthesis. According to [69], exogenous GAA stimulates insulin, a potent anticatabolic hormone that may stop the breakdown of protein in skeletal muscle. According to [70], broilers given GAA supplements presented noticeably increased levels of plasma insulin-like growth factor-1 (IGFI), an anabolic hormone that may promote muscle growth. Additionally, this group proposed that an increase in intramuscular creatine caused by GAA can draw water and expand the volume of muscle cells, meaning that highly hydrated muscles may promote protein synthesis and reduce protein breakdown [71]. The upregulation of genes linked to myogenesis (MYOG) and growth (IGFI and GH1) as well as the downregulation of MSTN, a gene encoding myostatin, a myokine that prevents muscle cell growth and differentiation, are additional mechanisms for GAA-stimulated muscle growth [59].

In the present study, the relative weights of the breast, leg, giblet (gizzard, liver, and heart), and immune organ (spleen, thymus, and bursa) weights were not influenced by HSD, which aligns with previous research [12, 50]. Moreover, no significant effect of GAA supplementation on the relative weight of the breast or leg was observed, as demonstrated by [72]. Additionally, no effect on gizzard, heart, or abdominal fat weight or immune organ weight was detected, whereas the relative liver weight was significantly reduced [73]. Oxidative stress affects hepatocyte proliferation, so supplementing broilers with antioxidants improves their liver antioxidant status and reduces their liver weight [74]. Therefore, the lower liver weight in the LSD-supplemented group could be attributed to the stronger antioxidant effects of the GAA supplements [75]. Furthermore, the GAA additive may enhance the liver function of broilers, potentially causing a decrease in liver enzymes in the present study.

Maintaining healthy animal immunity is crucial for performance and disease resistance, with antibody generation being a key component of the humoral immune defense mechanism [76]. As reported by [77], the immune status of poultry can be measured by determining the antibody response against foreign antigens such as Newcastle disease virus (NDV). Newcastle disease (ND) is on the list (A) of notifiable illnesses, and measuring the antibody titer against NDV is very important, as ND is a devastating avian infection that affects the poultry industry worldwide. Furthermore, mortality and trade losses caused by NDV cost the chicken industry millions of dollars each year [78, 79]. The present study revealed a significantly lower titer of antibodies against NDV in the HSD group than in the LSD group on day 21, which is consistent with previous findings [50]. The mechanism behind the immune response depression in HSD could be explained by the increased secretion of the CORT hormone [80]. With respect to the effect of GAA on the immune response, our results revealed that GAA supplementation improved the HI titer at 21 days. These results agreed with those of [81], who reported that antibody titers against NDV were significantly improved in creatine-treated groups of broiler chicks, and with those of [82], who reported that GAA supplementation in the diet of hens during the production period may have a significant effect on the immune response. These findings may be related to the decrease in CORT levels, which positively influence immune status. Similarly, a reduction in the CORT level and improvement in immune function were recorded in heat-stressed broilers supplemented with 0.6 g/kg GAA [83].

In the present research, neither SD nor GAA supplementation had a significant effect on the blood biochemical indices except for ALT and creatinine which were significantly lowered by dietary GAA supplementation. Additionally, the SD/GAA interaction lowered TAG levels. Many studies found that broilers under HSD did not cause any physiological adaptations to stress, with no significant changes in blood parameters [84]. For investigation of the GAA supplementation effect under LSD, it was shown that dietary GAA inclusion of up to 0.6% did not change blood biochemical parameters [85]. Furthermore, supplementing vegetable diets with 0.08% GAA or L-Arg for broilers subjected to heat stress did not impact blood biochemistry [86]. GAA supplementation levels (0.06, and 0.12%) were found to decrease triglyceride levels [87]. Nitric oxide production can be assessed by measuring the serum concentration of nitrite, which has roles in lipid metabolism [88]. In the present study, since nitrite levels were reduced by GAA supplementation, it is reasonable that triglycerides are also affected. Supplementing Tibetan pigs with 800mg/kg GAA was found to up-regulate the mRNA of the Adipose triglyceride lipase (ATGL) gene in the back fat [89]. Adipose ATGL is an essential enzyme that liberates fatty acids from triacylglycerol reserves [90] which could be another mechanism for GAA to lower TAG concentrations.

Liver enzymes (ALT and AST) are present in negligible concentrations but may increase due to damaged or diseased cells, indicating the status of liver function [91]. Creatinine is a byproduct of creatine phosphate metabolism, which the kidneys expel after skeletal muscles are used for energy production. As a result, it is used to assess kidney function [92, 93]. In the present study, the GAA-supplemented groups showed lower ALT activity and serum creatinine, which suggests the promoting effect of GAA on liver and kidney functions, respectively.

Serum or plasma biochemical profiles can indicate muscle damage due to disrupted sarcolemma integrity, leading to the leakage of enzymes like LDH and CK [94]. Our results showed that adding 0.6 g/kg GAA showed no significant effect on CK activity, while LDH activity was significantly increased. SD significantly impacted CK activity, whereas the LSD group displayed higher levels, but no significant effect on LDH. This finding can be explained by the fact that CK release is proportionate to exercise intensity and duration [95], as larger spaces allow birds to motivate their activities [96]. In agreement with our findings, a higher CK level was observed in birds from large cages which promote their movements than those from small cages [97]. 1.2 g/kg feed supplemental GAA to Arg-deficient diets did not affect CK levels [98]. GAA-supplemented groups showed increased LDH levels due to their ability to support rapid growth by providing muscles with ATP [99]. Rapid growth and competition among birds during rearing to obtain feed and drinking water can lead to muscle injury, which is observed in GAA-supplemented groups with high levels of LDH [100].

In the present study, serum CORT levels were studied as an indicator of physiological stress [101]. Our findings revealed that both LSD and GAA supplementation significantly reduced CORT levels. Consistent with these findings, plasma CORT levels were substantially lower in hens housed in floor pens with larger spaces [102]. Moreover, elevated levels of CORT have been observed in high SD broiler chickens [103]. Plasma CORT levels increased during the adaptive stress phase due to increased population density, causing birds to compete for feeding and watering space [104].

In different studies performed under various environmental conditions, consistent results have been obtained regarding the influence of GAA on CORT levels. Broilers fed diets supplemented with 1.2 g/kg feed GAA showed lower plasma CORT concentrations than those fed basal diets when subjected to a 3-h transport [105]. Birds grown under heat stress on the GAA-supplemented diet had significantly lower blood CORT levels than the non-supplemented group [26]. Under the circumstances of heat stress, the addition of 0.6 g/kg GAA has a positive effect on immunity by inhibiting the production of CORT. Although the precise mechanism by which GAA lowers CORT has not been identified, supplementing with GAA may lessen metabolic stress, or protein breakdown, by virtue of its arginine-sparing properties [26], and lessens the hypothalamus-pituitary-adrenocortical (HPA) axis's activation [106].

Oxidative stress can induce a deteriorated physiological status and oxidative damage to lipids, nucleic acids, and proteins in tissues [107, 108]. Living organisms can combat oxidative stress by producing antioxidant enzymes like SOD, GSH, and GPx, which are crucial for restoring the physiological system [109, 110]. In the current investigation, HSD induced oxidative stress in broilers by decreasing SOD, GPx, and catalase activity with no significant effect on the MDA and nitrite levels. In contrast, GAA supplementation augmented GPx and catalase activities and reduced the nitrite concentration with no significant effect on SOD and MDA levels. Our results were in line with most of the research that investigated the deleterious effect of HSD on broilers' antioxidant status [111, 112]. In a previous study, HSD reduced the pectoral muscle’s total antioxidant capacity and reduced the expression of antioxidant proteins such as liver catalase [113]. Furthermore, HSD has been shown to cause oxidative stress in broilers [54].

Previous studies reported that GAA may act as an antioxidant or a pro-oxidant agent in cellular systems depending on the rate and method of inclusion [114, 115]. Concerning the positive effect of GAA on antioxidant status; supplementation of broilers with 1200 mg/kg GAA increased liver GPx activity and decreased MDA serum levels in the cold-stressed environment [25]. Similarly, increases in GSH-Px and SOD activities were recorded in heat-stressed broilers fed 0.6 and 1.2 g/kg GAA supplementation [26]. GAA was effective in modulating the MDA rise and SOD reduction in the liver of broilers given Triiodothyronine (T3), (a model designed to enhance ascites syndrome, baseline metabolic rate, and to trigger mitochondrial-dependent reactive species formation) [116]. Many researchers concluded that because GAA can raise the body's level of creatine, it may be able to enhance the body's anti-oxidative capacity somewhat [117, 118]. Since creatine is found mainly in the skeletal muscles, it seems that the liver must handle a far higher burden of oxidative stress, which might originate locally or systemically and dietary GAA may help to mitigate this [119]. In addition to muscle creatine loading, there were other physiological functions of extra GAA such as insulin sensitizer and stimulator, γ-aminobutyric acid antagonist, and neuromodulation [120]. It was also accepted that GAA might function as a pro- and antioxidant. The result may be determined by all these interconnected physiological responsibilities, which might at least cause discrepancies in straightforward assessments of oxidative state [119].

Histomorphometric assessment of the three intestinal regions revealed that HSD significantly reduced the villus length and width of the ileum, as previously reported [12]. The primary location of nutrients absorption and the home of sizable bacterial populations is the ileum [121]. The impairment of intestinal villus growth is attributed to the subsequent stress on the birds reared under unfavorable overcrowding conditions [122]. In the poultry industry, broilers are subjected to harsh and stressful conditions when reared under HSD [14]. Such stress may induce dysfunction in the mucosal tight junctions [123], and the generation of cellular lipid peroxidation [124]. Additionally, increasing the stocking density to 39 kg/m^2^ has been reported to alter the composition of the ileal microbiota [125]. It has long been known that inactivity can lead to microbial dysbiosis in the human gut [126, 127]. As observed in our previous investigation, the mobility of broilers may be restricted by HSD [27]. Therefore, all these factors may negatively influence intestinal structure and epithelial development. Dietary inclusion of GAA had no significant effect on intestinal morphometric parameters under both LSD and HSD conditions. In a previous study, no significant change in the gut morphometric characteristics of birds fed 0.6 or 1.2 g/kg GAA was detected [128].

## Conclusion

HSD negatively affects the performance, ileal histomorphometry, immune and antioxidant status of broilers. The inclusion of 0.6 g/kg GAA improved the BW, BWG, FCR, EPEF, and dressing percentage. Additionally, GAA supplementation enhanced the immune status of the broilers by increasing the titer of antibodies against NDV. GAA reduced stress in the HSD groups by lowering CORT levels and improving their antioxidant status. Thus, the dietary use of GAA as a beneficial additive may offer a nutritional strategy in broiler farming to overcome the deleterious effects of HSD stressors. Further studies are needed to investigate the effects of GAA supplementation on skeletal muscle creatine, ATP levels and meat quality under HSD conditions. Although there are various ways in which GAA can stimulate the growth of skeletal muscle, the precise role that each mechanism plays in either promoting net muscle growth or preventing muscle loss is still unknown. Additionally, there is currently no information available regarding any potential interactions between distinct pathways, whether they are antagonistic, neutral, or synergistic. Moreover, studies on the effects of GAA supplementation on gut health and the microbiota under stressful conditions should be expanded.

## Data Availability

All data generated or analyzed during this study are included in this published article.

## Abbreviations

ADG: Average daily gain
BW: Body weight
BWG: Body weight gain
CK: Creatine kinase
CORT: Corticosterone
EPEF: European Performance Efficiency Factor
FC: Feed consumption
FCR: Feed conversion ratio
GPX: Glutathione peroxidase
GSH: Glutathione (reduced form)
LDH: Lactate dehydrogenase
MDA: Malondialdehyde
SOD: Superoxide dismutase
TAC: Total antioxidant capacity
WFI: Weekly feed intake

## Declarations

### Prior publication

Data have not been published previously.
